# Annual Migration of *Agrotis segetum* (Lepidoptera: Noctuidae): Observed on a Small Isolated Island in Northern China

**DOI:** 10.1371/journal.pone.0131639

**Published:** 2015-06-26

**Authors:** Jianglong Guo, Xiaowei Fu, Xiao Wu, Xincheng Zhao, Kongming Wu

**Affiliations:** 1 State Key Laboratory for Biology of Plant Diseases and Insect Pests, Institute of Plant Protection, Chinese Academy of Agricultural Sciences, Beijing, China; 2 Department of Entomology, College of Plant Protection, Henan Agricultural University, Zhengzhou, China; CNRS, FRANCE

## Abstract

Migration behavior of the turnip moth, *Agrotis segetum* (Lepidoptera: Noctuidae), is not well known by far. Here, we present the data from an 11-year study on *A*. *segetum* by means of searchlight trapping and ovarian dissection on Beihuang (BH) Island, which located in the center of the Bohai Strait in northern China. The data showed a large number of *A*. *segetum* flight across the strait each year, which provides direct evidence that *A*. *segetum* is a long-distance migrant, migrating at least 40 - 60 km to reach the trapping site. The migration period during 2003-2013 ranged from 115 to 172 d. Among the catches, the proportion of females was significantly higher than that of males in each month from May to September. Ovarian dissection showed that the proportion of mated females and the proportion of sexually mature females was significantly higher than that of unmated females and sexually immature females in early summer, respectively, but conversely in autumn. The early summer populations migrate in a south-north direction, which might undertake a long-distance flight on several successive nights. The autumn populations migrate in a north-south direction, which might originate not far from the trapping site. Based on these findings, the migratory physiology of *A*. *segetum* was discussed.

## Introduction

The turnip moth, *Agrotis segetum* Denis and Schiffermaller (Lepidoptera: Noctuidae), is one of the most important crop pests which distributed widely in Europe, Asia and Africa [1–4]. *A*. *segetum* larvae, also called cutworms, are a kind of soil pest damaging crops by feeding or ‘cutting’ the stems of seedlings at or below the growing point. Many important crops and vegetables, such as wheat, corn, potato, pea, sorghum, cabbage, and beet, can be infested by *A*. *segetum* [5–8]. In order to develop effective management strategies on *A*. *segetum*, many studies have focused on monitoring and biological control, including investigations using traps, pathogenic viruses and bacteria, [6,9–17].

Migration, a seasonal to and from movement of insect populations between regions where conditions are alternately favorable or unfavorable (involves adaptations for escaping from ecological and environmental stresses, i.e. climatic conditions, food sources, etc.), is an important reason for crop pest sudden outbreaks [18–21]. Well-understanding of insect migration facilitates the development of forecasting systems, and enables the design of sound management strategies. On the one hand, it is commonly accepted that the widely distribution of *A*. *segetum* due to the strong potential to undertake long-distance migration, and recently radar observations also confirmed that [22]. However, it is still unclear (1) whether the migration of this species is a regular ecological behavior, and if so, (2) what pattern of seasonal migration this species exhibits in case of a regular ecological behavior. On the other hand, the ‘oogenesis-flight syndrome’, namely the onset of oogenesis causes a cessation of migratory behavior, was first noted by Kennedy [23] and thoroughly reviewed and defined as a general principle by Johnson [18, 24]. The principle of oogenesis-flight syndrome has been widely accepted and used as model in migration research. Whether the migration of *A*. *segetum* is bound by the ‘oogenesis-flight syndrome’ remains unknown.

The data presented here are the results from a long-term investigation on seasonal migration of *A*. *segetum* across the sea, which was carried out by means of searchlight trapping and ovarian dissection on a small isolated island, Beihuang (BH), located in the center of the Bohai strait in northern China. BH, as well as its neighbor islands, is an ideal site for investigating insect migration because (1) it is a long distance far away from the mainland, about 60 km to the south and 40 km to the north; (2) no arable lands and no original agricultural insect pests are present. The findings of this study can improve our knowledge for understanding the migration behavior of *A*. *segetum*, as well as its population and evolutionary ecology. It is also helpful for us to develop more effective management strategies against this pest species.

## Materials and Methods

### Location and trapping techniques

**Fig 1:**
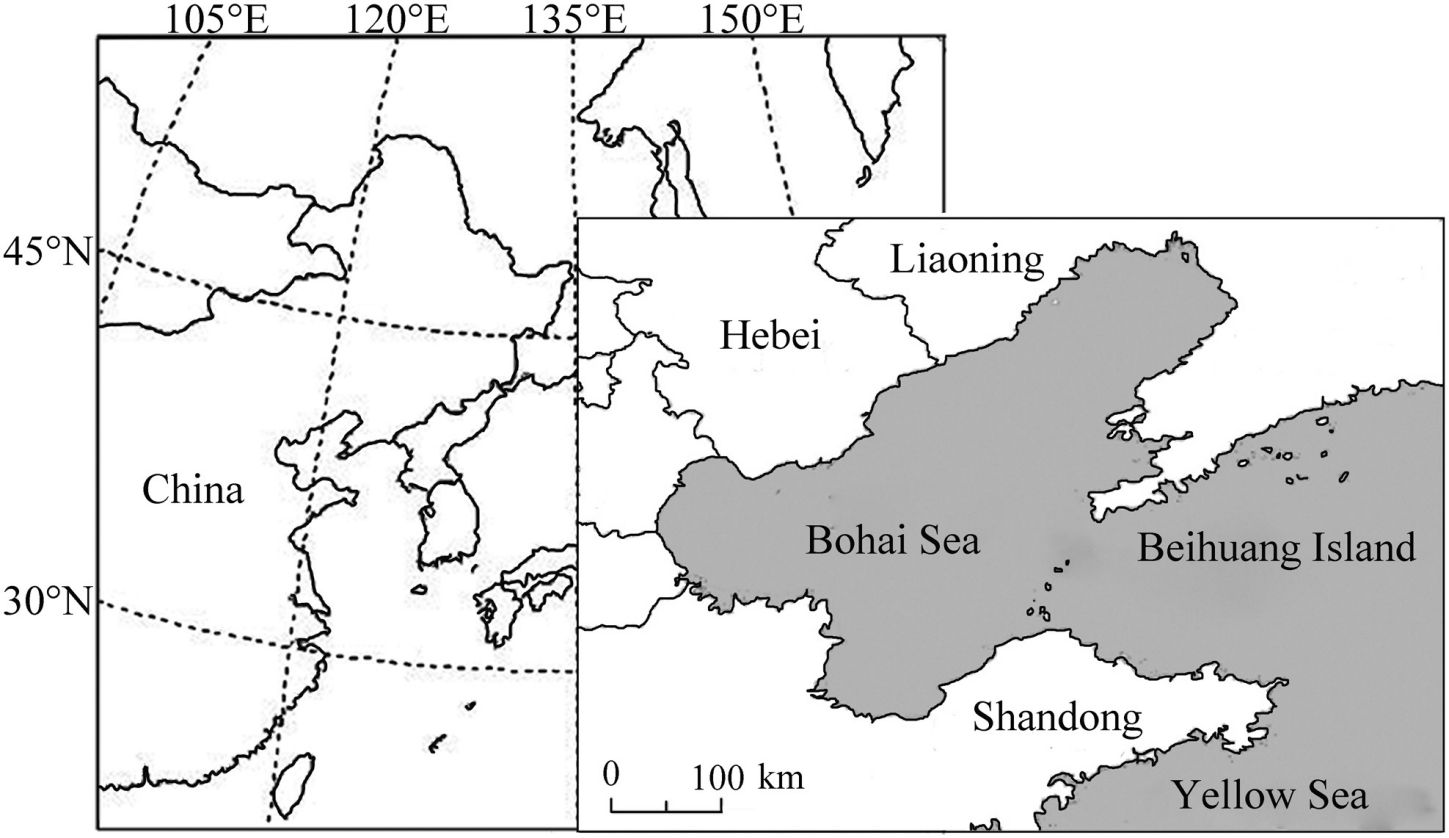
Maps showing the zone of East Asia and the position of Beihuang (BH) Island, the searchlight trapping site (right-hand map), relative to the Bohai Sea and Yellow Sea.

Searchlight trapping was carried out from April to October during 2003–2013 on BH (38°24´N; 120°55´E). BH is a small island with ~ 2.5 km^2^ area, located in the center of the Bohai Strait, at a distance of ~ 60 km from the mainland to the south and ~ 40 km to the north (~ 2.5 km from the neighbor Nanhuang Island) (Fig 1). As the Chinese Academy of Agricultural Sciences is an affiliated part of the Ministry of Agriculture of the People’s Republic of China, no specific permits were required for this study. No endangered or protected species were involved in this study. A vertical-pointing searchlight trap positioned on a platform ~8 m above sea level (asl), was used to attract and capture high-altitude migrants (up to ~500 m asl) [25]. The trap was equipped with a 1,000 W metal halide lamp (model JLZ1000BT; Shanghai Yaming Lighting Co. Ltd., Shanghai, China), that produces a vertical beam of light with a luminous flux of 105,000 lm, a color temperature of 4,000 K, and a color rendering index of 65 [26].

The searchlight trap was switched on at sunset and switched off at sunrise on all nights during the period of study, with the exception of days that exhibited power cuts or when heavy rain occurred. Trapped insects were collected with a nylon net bag (60 mesh) beneath the trap, which was changed manually every 2 h each night. The trapped insects were kept in a freezer at -20°C for 4 h before being identified. To confirm whether there is local population of *A*. *segetum* on BH, visual observations were carried out daily to detect *A*. *segetum* larvae on any potential wild host plants from spring to autumn during 2009–2013.

### Ovarian dissection

To test the hypothesis of ‘oogenesis-flight syndrome’, a subsample of 20 *A*. *segetum* females (or all individuals if the total capture was < 20), were randomly selected from each night’s catch, and were dissected under a stereomicroscope (model JNOEC-Jsz4; Motic China Group Co.Ltd., Xiamen, China) to determine the development level of the ovary from 2010–2013. The level of ovarian development (level 1–5) was estimated according to the criteria described by Li et al. [27] and Qi et al. [28]. The females with ovarian development level 1 and level 2 were regarded as “sexually immature individuals”, and those with level 3–5 were regarded as “sexually mature individuals” [27,28]. The average monthly level of ovarian development was calculated as the sum of individual levels of ovarian development divided by the number of females dissected. The presence and number of spermatophores in the female spermatheca was used to determine the mating occurrence of *A*. *segetum* [29]. The proportion of mated females is calculated by the number of mated females divided by the number of dissected females.

### Data analysis

All collected *A*. *segetum* were trapped at night. Date of trapping indicates the period from sunset of that day to sunrise of the next day. In order to show the annual pattern of migration, the nightly catches of each year, as well as the nightly mean catches of 2003–2013 were presented. In addition, the proportion of mated females and the proportion of sexually matured females for each night were calculated. The data were subject to one-way analysis of variance (ANOVA), with month as the fixed effect and year as the random effect (PROC ANOVA) to determine the effect of time. If the ANOVA indicated a significant difference between months, Tukey's HSD (honestly significant difference) test was followed to separate the means. Differences of the mean proportion of females and males (i.e. sex ratio, females: males) in each month from 2010 to 2013 with the ratio of 1:1 were compared by using chi-square test (PROC FREQ). Due to the lack or incomplete of data of the sex ratio, the data of 2003–2009 and 2014 were not included for analysis. Except the analysis of the sex ratio with SAS software [30], other statistical analyzes were performed by SPSS software [31].

## Results

### Annual and monthly pattern of migration

**Fig 2:**
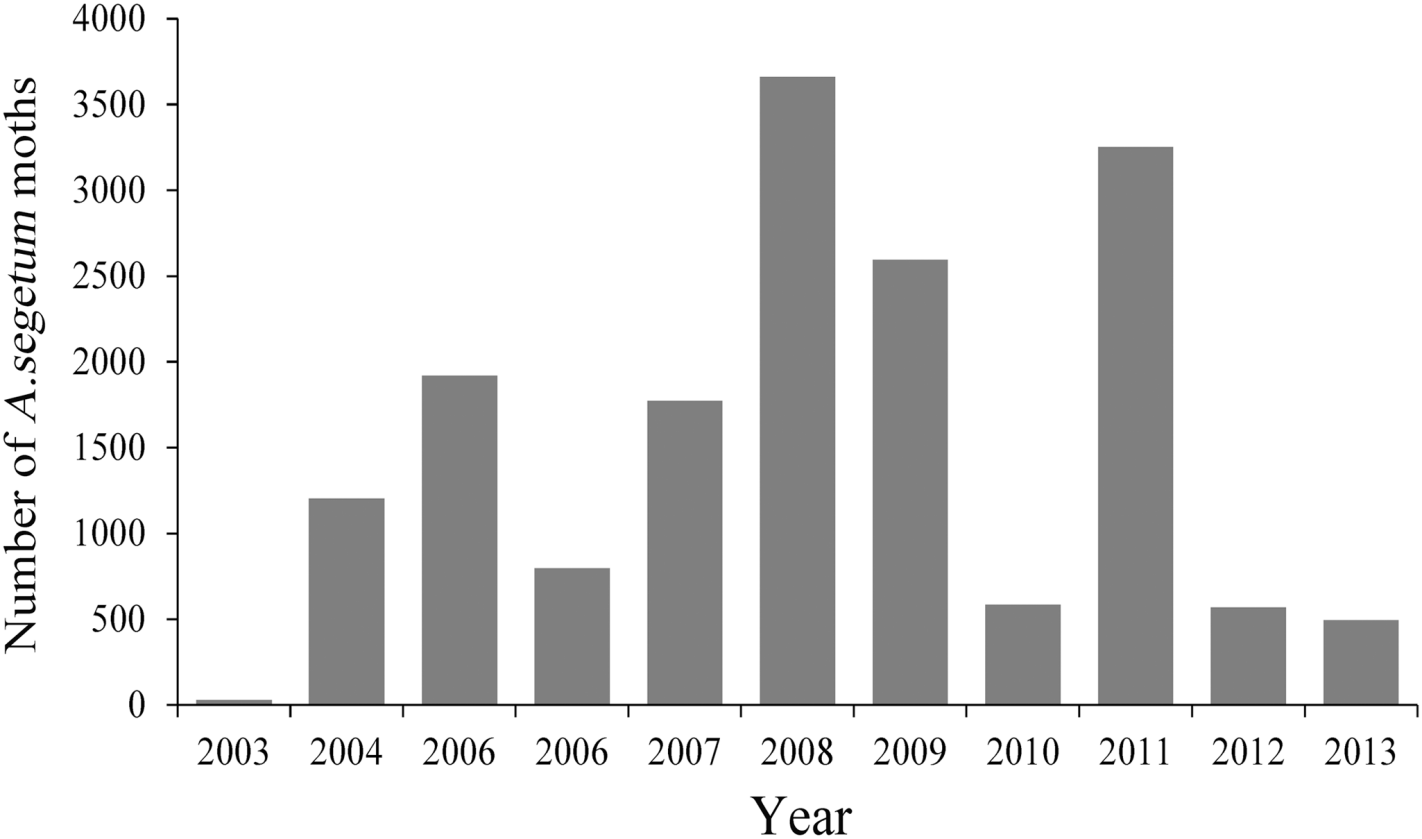
Annual catches of *A*. *segetum* in the searchlight trap on BH from 2003 to 2013.

During the study period of 2003–2013, no *A*. *segetum* larvae were found on BH. However *A*. *segetum* adults were regularly captured in the searchlight trap, strongly suggesting that *A*. *segetum* moths immigrate from the mainland rather than emerging locally. They migrated at least 40 to 60 km to reach the trapping site across the sea. The total number of captured *A*. *segetum* individuals during the 11-year period was 16875, and for each year the number ranged from 494 to 3660, except for 27 individuals in 2003 (Fig 2).

**Fig 3:**
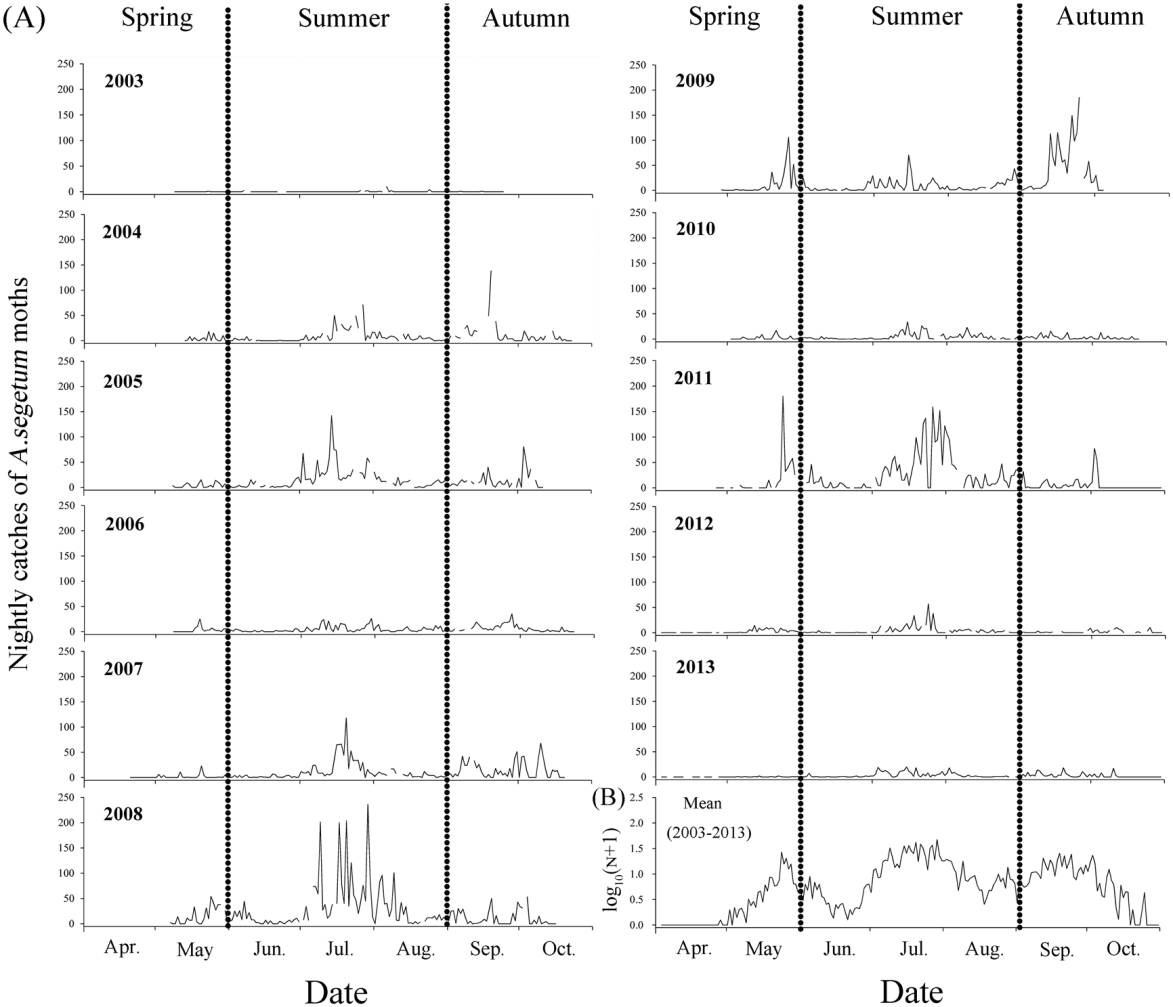
Nightly catches (A) and mean logarithm of numbers in the 11 years (B) of *A*. *segetum* in the searchlight trap on BH from April to October.

Besides the annual fluctuations in the number of *A*. *segetum* moths, we also observed considerable variation in months from May to September. Light trapping indicated that there were three noticeable peak periods of migration over the sea, namely, in late-May, early- and late-July, mid-September to early-October (Fig 3). There were no catches in late-October (Fig 3). There was significant inter-month difference (*F* = 4.46, *df*_1_ = 6, *df*_2_ = 63, *P* = 0.001) in the nightly mean catches of *A*. *segetum* in the 11 years (Fig 3).

The first capture of *A*. *segetum* generally occurred in late-April and early-May and the latest generally in late-October. The mean time from the earliest trapping to the latest trapping of *A*. *segetum* within a year is 156±5 d, and the shortest time span is 115 d in 2003 and the longest 172 d in 2012 (Table 1).

**Table 1:** Dates of first and final capture of *A*. *segetum* and duration of capture period in the eleven years on Beihuang Island in northern China.

Year	Date of first capture (n)ª	Date of final capture (n)ª	Duration of capture(d)	Date of peak catches (n)ª
2003	23 May (1)	15 September (1)	115	06 August (10)
2004	14 May (1)	20 October(2)	159	19 September (138)
2005	08 May (3)	08 October (14)	153	14 July (142
2006	17 May (7)	20 October (2)	156	27 September (35)
2007	02 May (6)	17 October (14)	168	20 July (118)
2008	07 May (2)	12 October (4)	158	29 July (236)
2009	28 April (1)	01 October (30)	156	24 September (185)
2010	07 May (4)	17 October (5)	163	15 July (34)
2011	06 May (5)	03 October (55)	150	24 May (180)
2012	06 May (1)	25 October (10)	172	24 July (57)
2013	05 May (1)	11 October (3)	159	15 July (20)

^ª^ The numbers of *A*. *segetum* moths captured are given in parentheses next to the date of capture.

### Sex ratio, proportion of mated females, and ovarian development

**Fig 4:**
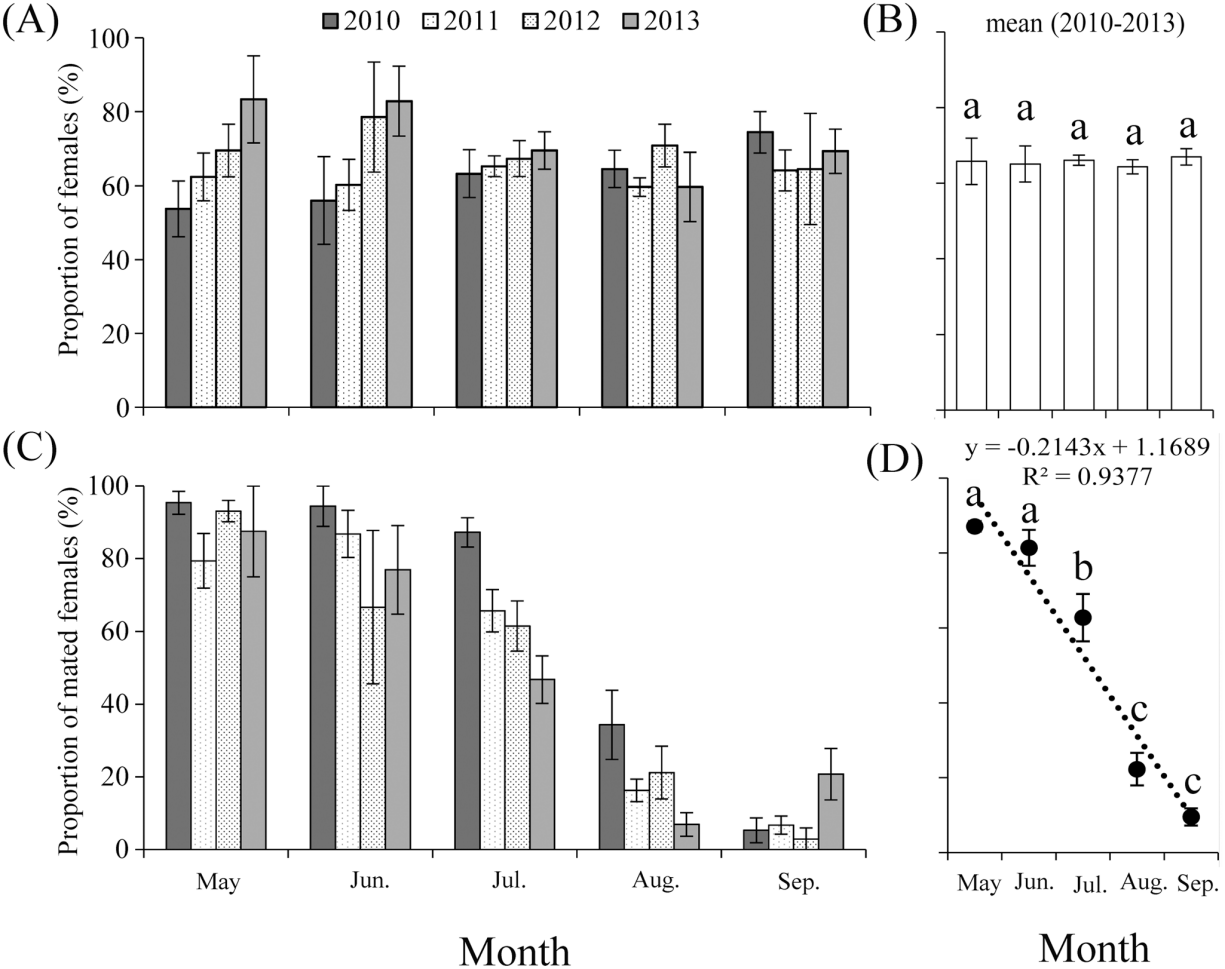
Proportion of females (A-B) and proportion of mated females (C-D) of *A*. *segetum* captured in the searchlight trap on BH during 2010–2013. The histograms in A and C indicate mean daily proportion in each month, and the error bars represent standard errors between days in that month. The histograms in B and the lines in D indicate mean proportion of females and mated females from 2010–2013 in each month, and the error bars represent standard errors between years in that month. Bars sharing the same letter indicate that there were no significant inter-month differences at the 5% level by Tukey's HSD tests. Few moths were trapped in April and October, so these months are not presented and the same below. Linear model (dotted lines): *y* = -0.2143 *x* + 1.1689; *R* ^2^ = 0.9377.

From May to September 2010–2013, the vast majority of trapped *A*. *segetum* were females and chi-square tests showed that the sex ratio (females: males, monthly sum of catches) was significantly higher than 1:1 in all months: May, χ^2^ = 25.24, *df* = 1, *P* < 0.001; June, χ^2^ = 15.13, *df* = 1, *P* < 0.001; July, χ^2^ = 187.14, *df* = 1, *P* < 0.001; August, χ^2^ = 58.59, *df* = 1, *P* < 0.001; September, χ^2^ = 57.07, *df* = 1, *P* < 0.001. However, there was no significant inter-month difference (*F* = 0.07, *df*_1_ = 4, *df*_2_ = 15, *P* = 0.99) in the mean proportion of females from 2010–2013 (Fig 4A and 4B).

**Fig 5:**
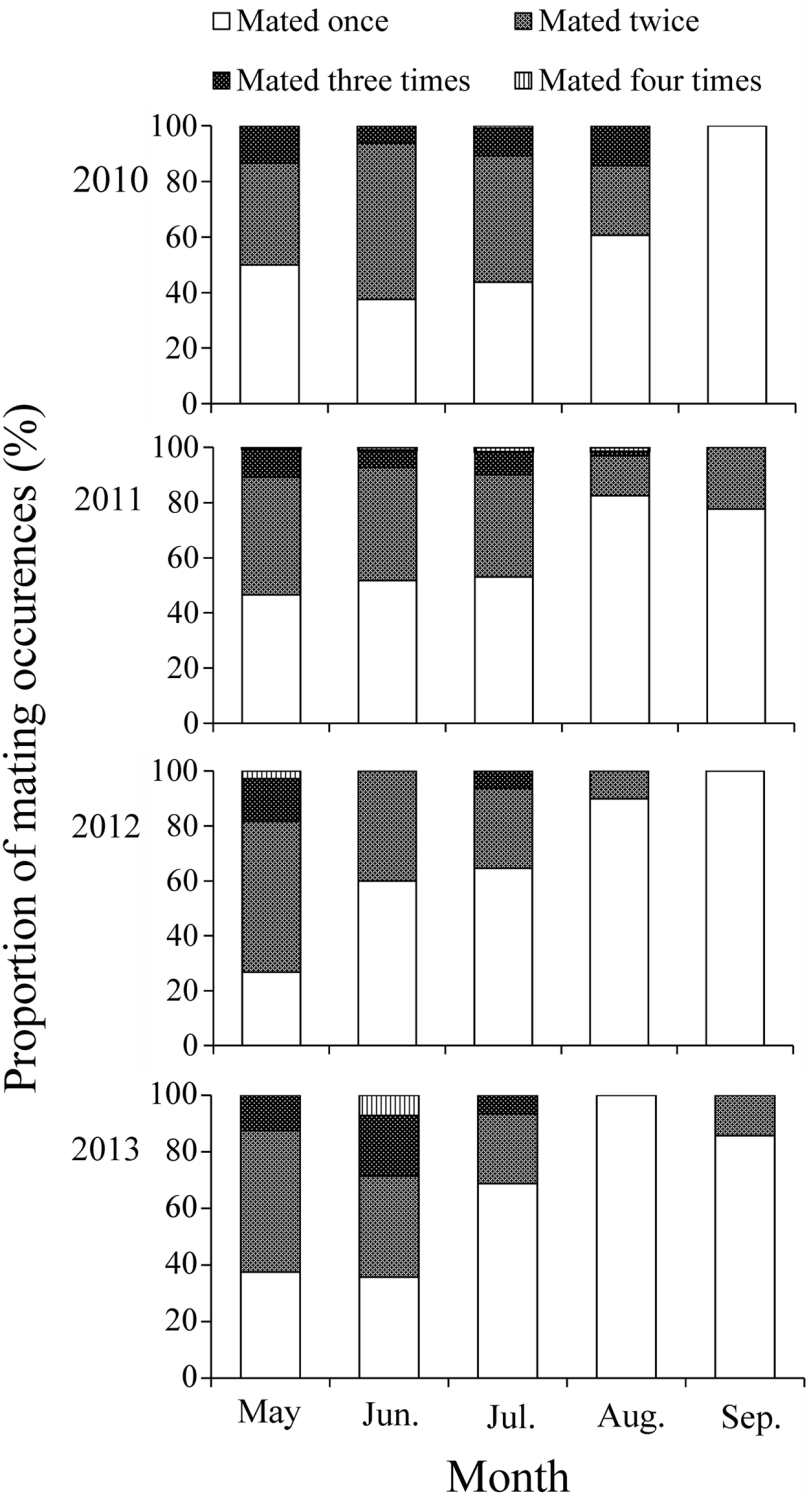
Proportion of mating occurrences of *A*. *segetum* females captured in the searchlight trap on BH during 2010–2013.

The proportions of mated females with respect to all dissected females, however, varied considerably between months (*F* = 57.51, *df*_1_ = 4, *df*_2_ = 15, *P* < 0.001) (Fig 4C and 4D). The proportion of mated females in May and June reached 87.11 ± 1.37%, 81.38 ± 4.79%, respectively, which were significantly (May: *t* = 20.49, *df* = 6, *P* < 0.001; June: *t* = 5.15, *df* = 6, *P* = 0.014) higher than the mean proportion of unmated individuals (Fig 4C and 4D). The proportion of mated females in July was 62.72 ± 6.33% and was not significantly different (*t* = 1.95, *df* = 6, *P* = 0.15) from the mean proportion of unmated individuals. In August and September, however, the proportion of mated females declined rapidly and dropped to 22.28 ± 4.33%, 9.52 ± 2.30%, respectively, which was significantly (August: *t* = -5.96, *df* = 6, *P* = 0.009; September: *t* = -12.42, *df* = 6, *P* = 0.001) lower than the mean proportion of unmated individuals (Fig 4C). Thus, the proportion of mated females has a significant falling trend from May to September during the 11 years (linear model, *y* = -0.2143*x* + 1.1689; *R* ^2^ = 0.9377, *F* = 45.13, *P* = 0.007) (Fig 4D). Among the mated females, the vast majority just mated once, some mated twice, and only a few mated three and four times in the first three months (May, June, and July). Most of mated females mated once in August and September (Fig 5).

**Fig 6:**
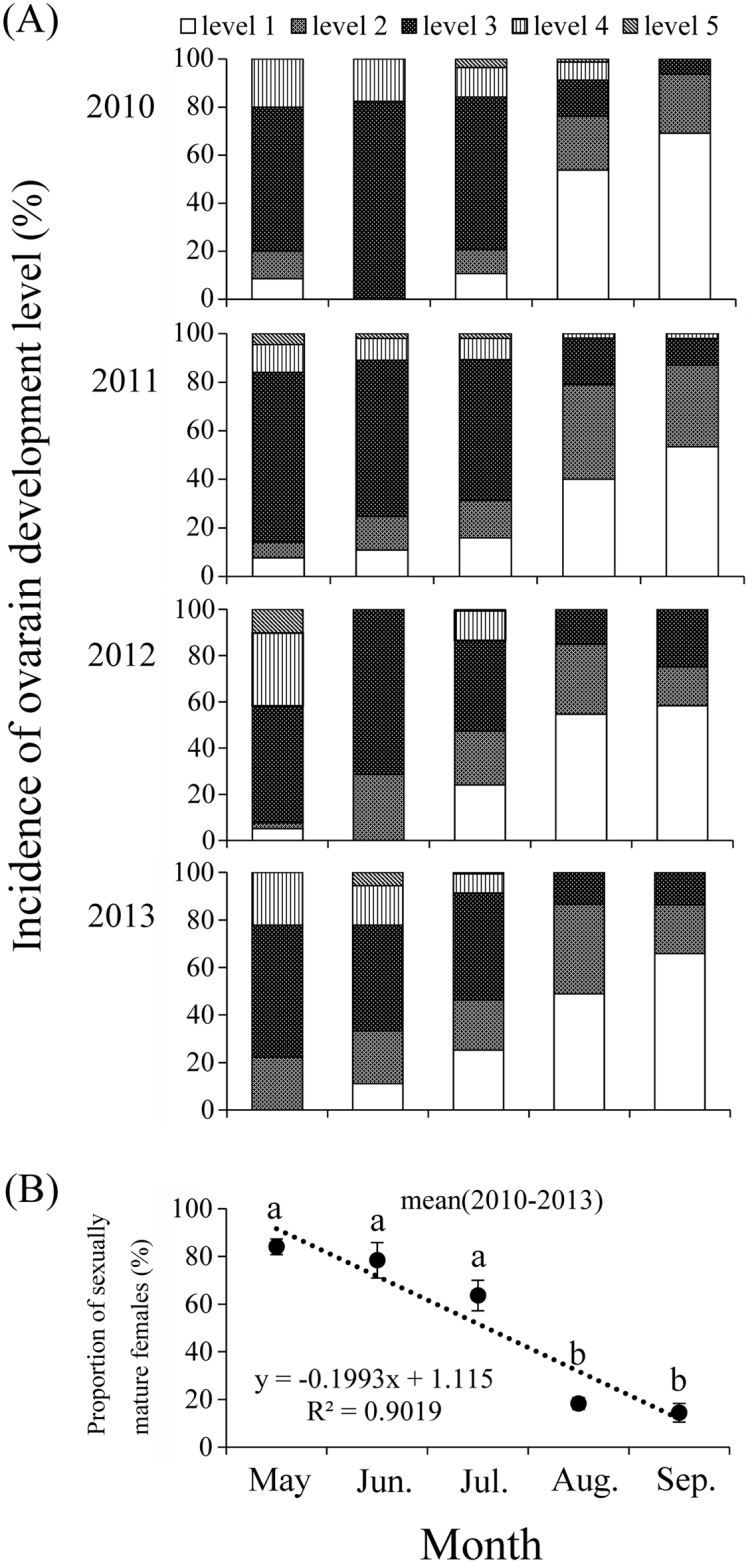
Incidence of ovarian development (A) and mean proportion of sexually mature females (B) of *A*. *segetum* captured in the searchlight trap on BH during 2010–2013. The lines in B indicate average proportion of sexually mature females from 2010 to 2013 in each month, and the error bars represent standard errors between years in that month. Bars sharing the same letter indicate that there were no significant inter-month differences at the 5% level by Tukey´s HSD tests. Linear model (dotted lines): y = -0.1993x + 1.115; R^2^ = 0.9019.

The ovaries of captured females from May to September show different levels of development (Fig 6A). The proportion of sexually immature females with the ovaries at the development level 1 and 2 exhibited an upward trend from May to September during 2010–2013 (Fig 6A). In other words, the proportion of sexually mature females (ovarian development levels 3–5) showed an apparent downward trend from May to September (linear model, *y* = 0.1993*x* + 1.115; *R* ^2^ = 0.9019, *F* = 27.59, *P* = 0.013) (Fig 6B). In May, most of *A*. *segetum* females showed a certain degree of ovarian development, at the levels 3–5, and the mean proportion of sexually mature females reached 84.02 ± 3.28%, which was significant (*t* = 8.02, *df* = 6, *P* = 0.004) higher than the mean proportion of sexually immature females. The mean proportions of sexually mature females in June and July reached 78.34 ± 7.43%, 63.58 ± 6.41%, respectively, which was not significantly different (June: *t* = 2.52, *df* = 6, *P* = 0.086; June: *t* = 2.08, *df* = 6, *P* = 0.130) from the mean proportion of sexually immature females. However, the proportion of sexually mature females declined rapidly and dropped to 18.28 ± 2.44%, 14.42 ± 3.90% in August and September, respectively, which was significantly (August: *t* = -10.91, *df* = 6, *P* = 0.002; September: *t* = -7.26, *df* = 6, *P* = 0.005) lower than the mean proportion of sexually immature (Fig 6B).

## Discussion

During 11 years of searchlight trapping study on BH provided direct evidence that *A*. *segetum* is a long-distance migrant (which flight at least 40–60 km across the Bohai Strait), because no host crops or larvae of this species were found on this small isolated island. The trapping results also suggested that the migration of *A*. *segetum* is a regular ecological behavior, and not an occasional phenomenon. The migratory populations of *A*. *segetum* showed significant seasonal oscillations, which might be related to the population size in their original areas. A similar phenomenon was also observed in previous studies of other migratory species, such as the beet armyworm *Spodoptera exigua*, *H*. *armigera*, the meadow moth *Loxostege sticticalis*, the yellow dragonfly *Pantala flavescens*, *Apolygus lucorum*, and *P*. *xylostella* [25,32–41].

The catches of *A*. *segetum* include both females and males, but their ratio is biased, i.e. the proportion of females was significantly higher than that of males in each month from May to September 2010–2013. The different catches between females and males may indicate that *A*. *segetum* females have greater flight capability than males or females are more attracted to light because many insect species have noticeable sexual differences in the behavior of flight-to-light [42]. Meanwhile, it had been demonstrated that the flight performance of insects is affected by many factors, including gender [19,43]. To verify the sexual difference in the flight performance and sensitivity to light of *A*, *segetum*, further flight mill experiments or other related experiments are needed.

Migratory behavior in insects correlates with reproductive development in females, i.e. migration often occurs during the pre-reproductive stage of adults (the primary stage of ovarian development) [18,24,44,45]. Unexpectedly, the results of dissecting females clearly showed that mated and sexually mature females of *A*. *segetum* predominated in early summer. It suggests that the migration of *A*. *segetum* is not inhibited by the developed ovary and mating, in other words, the migration is not in line with the hypothesis of oogenesis-flight syndrome. Similar results were also observed in *P*. *xylostella* and armyworm *Mythimna separata* [38,46]. On the contrary, *A*. *segetum* captured in September and October were significantly lower in the proportion of sexually mature females than in other months. This finding is consistent with the viewpoint of migration initiated by sexually immature individuals [23,44,47–49]. The migratory events of the same species show different patterns in different seasons.

The seasonal differences in the reproductive status of migratory females of *A*. *segetum* could be explained by the different flight distance and the temperature variation between seasons. In early summer, *A*. *segetum* migrates in the direction from the south to the north. The catches of *A*. *segetum* in the trapping site might have undertaken a long-distance flight on several successive nights [38]. During this period, the ovary development and mating behavior occurred. While, in autumn, *A*. *segetum* migrating in the direction from the north to the south, originates not far away from the trapping site. In addition, the different temperature of summer and autumn exhibit effects on the development of the ovary. In *A*. *ipsilon*, the development of the ovaries is slower in autumn than in spring [49]. Temperature is an important factor affecting ovary development via regulating the release of juvenile hormone. The lower temperature, plus short photoperiod inhibits the synthesis and secretion of JH.

The current study provides a direct evidence that *A*. *segetum* can migrate across the Bohai Strait, thus the hypothesis that *A*. *segetum* is a migratory species is confirmed. These findings are essential for the development of forecasting systems of spread. Despite the fact that the exact origin area of trapped *A*. *segetum* in this study is still unclear, it may be likely that the migration path way of this species was similar to that of other noctuid species in the same site and situation [25,35–37], which migrate toward the north in prevailing southerly winds during spring and summer, and return back in prevailing northerly winds during autumn. In order to make more precise prediction, studies are needed to clarify the migration trajectories by using a variety of methods, such as detecting insecticide resistance, tracing elements in the insect’s body, and genetic diversity [50–53].
